# A community-based participatory approach and engagement process creates culturally appropriate and community informed pandemic plans after the 2009 H1N1 influenza pandemic: remote and isolated First Nations communities of sub-arctic Ontario, Canada

**DOI:** 10.1186/1471-2458-12-268

**Published:** 2012-04-03

**Authors:** Nadia A Charania, Leonard JS Tsuji

**Affiliations:** 1 Department of Environment and Resource Studies, University of Waterloo, Waterloo, ON, Canada

## Abstract

**Background:**

Public health emergencies have the potential to disproportionately impact disadvantaged populations due to pre-established social and economic inequalities. Internationally, prior to the 2009 H1N1 influenza pandemic, existing pandemic plans were created with limited public consultation; therefore, the unique needs and characteristics of some First Nations communities may not be ethically and adequately addressed. Engaging the public in pandemic planning can provide vital information regarding local values and beliefs that may ultimately lead to increased acceptability, feasibility, and implementation of pandemic plans. Thus, the objective of the present study was to elicit and address First Nations community members’ suggested modifications to their community-level pandemic plans after the 2009 H1N1 influenza pandemic.

**Methods:**

The study area included three remote and isolated First Nations communities located in sub-arctic Ontario, Canada. A community-based participatory approach and community engagement process (i.e., semi-directed interviews (n = 13), unstructured interviews (n = 4), and meetings (n = 27)) were employed. Participants were purposively sampled and represented various community stakeholders (e.g., local government, health care, clergy, education, etc.) involved in the community’s pandemic response. Collected data were manually transcribed and coded using deductive and inductive thematic analysis. The data subsequently informed the modification of the community-level pandemic plans.

**Results:**

The primary modifications incorporated in the community-level pandemic plans involved adding community-specific detail. For example, ‘supplies’ emerged as an additional category of pandemic preparedness and response, since including details about supplies and resources was important due to the geographical remoteness of the study communities. Furthermore, it was important to add details of how, when, where, and who was responsible for implementing recommendations outlined in the pandemic plans. Additionally, the roles and responsibilities of the involved organizations were further clarified.

**Conclusions:**

Our results illustrate the importance of engaging the public, especially First Nations, in pandemic planning to address local perspectives. The community engagement process used was successful in incorporating community-based input to create up-to-date and culturally-appropriate community-level pandemic plans. Since these pandemic plans are dynamic in nature, we recommend that the plans are continuously updated to address the communities’ evolving needs. It is hoped that these modified plans will lead to an improved pandemic response capacity and health outcomes, during the next public health emergency, for these remote and isolated First Nations communities. Furthermore, the suggested modifications presented in this paper may help inform updates to the community-level pandemic plans of other similar communities.

## Background

Public health emergencies, such as an influenza pandemic, have the ability to cause high morbidity and mortality rates in humans [1]. Research indicates that disadvantaged populations will be disproportionately affected by an influenza pandemic, thereby exacerbating previously established social and economic inequalities [2,3]. For instance, some First Nations communities suffer from conditions of overcrowded housing and extreme poverty, in addition to inadequate access to many of the amenities of life (e.g., running water, health care, etc.) [4-6]. Geographically remote (i.e., nearest service center that provides access to Government of Canada’s programs and services with year-round road access is located over 350 kilometers away) and isolated (i.e., only accessible by planes year-round) First Nations communities typically face additional challenges, such as, limited transportation of required supplies and resources (especially during harsh weather situations) and continuous shortages of health care personnel [7-10]. Indeed, Canada’s First Nations, especially populations living in geographically remote communities, were severely impacted by the recent 2009 H1N1 influenza pandemic (pH1N1) [6,11-13]. During the pH1N1 response, some remote and isolated First Nations communities reported problems, such as, confusion and lack of preparedness, owing to ill-defined roles and responsibilities of government bodies overseeing the delivery of health care and insufficient details in community-level pandemic plans [14].

Another public health emergency is inevitable [15]; therefore, countries worldwide are encouraged to have effective pandemic plans in place to minimize the associated social and economic disruption [2,16]. Typically, national pandemic plans around the world have involved limited public consultation, and instead have been heavily guided by government and public health agencies, and panels of expert scientists [17-20]. However, it is important that recommended actions in pandemic plans are accepted by the public and can be realistically executed at the community level [20]. Research has shown that public engagement in pandemic planning can aid in understanding community perspectives and local values [21,22].

Furthermore, since all individuals are affected by an influenza pandemic, a societal approach to pandemic planning is recommended [2,17,22]. Unfortunately, various disadvantaged populations have not been sufficiently involved in the pandemic planning process [23-26]. For instance, Canada’s Assembly of First Nations noted that First Nations had not been sufficiently involved in the development of federal and provincial pandemic plans to date [27]. Ethically addressing the needs of disadvantaged populations should be a cornerstone of pandemic planning [3]. Disadvantaged populations best understand how they will be affected by a public health emergency and are able to identify barriers to current public health recommendations, placing them in a position to create innovative mitigation strategies [23]. Thus, engaging the public, especially disadvantaged populations, can aid in providing pandemic policy planners with information about the unique, local issues they face, which may lead to more successful implementation of pandemic plans and potentially mitigate the inequity that may occur during an influenza pandemic [5,22,23].

Studies have shown that it is important for research findings to be promptly and optimally utilized to change current practice, thus enhancing the knowledge translation process of linking research to action [28-31]. In Canada, the Canadian Institutes of Health Research defines the term knowledge translation as, “a dynamic and iterative process that includes synthesis, dissemination, exchange and ethically-sound application of knowledge to improve the health of Canadians, provide more effective health services and products and strengthen the health care system” [32]. The present study is an example of knowledge translation where qualitative information was collected from participants residing in three remote and isolated First Nations communities of sub-arctic Ontario, Canada, and used to modify the existing community-level pandemic plans. A knowledge translation approach was appropriate for this study, as it aimed to bridge the knowledge-to-action gap regarding how to engage disadvantaged populations in the pandemic planning process [28,30]. The purpose of this paper is to describe the community-based participatory approach and community engagement process used, and to highlight the resulting evolutionary stages of each community’s pandemic plan.

## Methods

### Community-based participatory approach

Three remote and isolated First Nations communities were included in the present study since residents had verbally expressed an interest in modifying their community-level pandemic plans to better address their unique living conditions during the next public health emergency. A community-based participatory approach was used as participatory research methods have been shown to be successful when working with Aboriginal communities [33-35]. This approach values collaboration with the community throughout the research process [33]. Thus, a community-based advisory group was formed of representatives from the communities’ health centers and Band Councils (i.e., local First Nations governing body formed of elected community members) and they played a large, collaborative role in designing the study, approving the interview questions, validating the results, and disseminating the study’s findings [14,36]. Additionally, research participants were actively involved and engaged throughout the process of modifying their respective community’s pandemic plan. Ethics approval for this research was granted by the University of Waterloo’s Office of Research Ethics.

### Study area and characteristics

The three study communities (referred to as Community A, B, and C for anonymity purposes) are remote and isolated First Nations communities located in northern Ontario, Canada; thus, the communities’ governmental organizational structure (i.e., local, provincial, and federal) responsible for the delivery of health services during a public health emergency were similar [37]. The Band Council of each study community plays a large role in making decisions regarding how health care is delivered [37]. Also, each community has a federally-funded health center, which provides community public health care and education [37]. Acute and/or chronic primary health care is provided by nurses working in the extended role at either a federally-funded nursing clinic or provincially-funded hospital [37].

### Study population, data collection, and analyses

**Figure 1:**
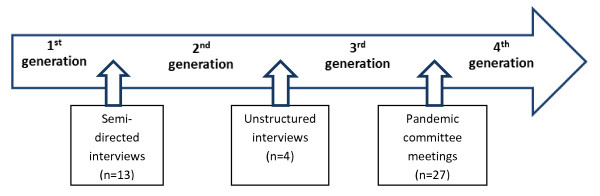
Stages of modifying community-level pandemic plans of three remote and isolated First Nations communities.

Each study community had a community-level pandemic plan in place prior to pH1N1, which will be referred herein as the 1^st^ generation pandemic plan. Being ethically and culturally appropriate for the region, informed verbal consent was obtained from participants prior to conducting the semi-directed interviews, unstructured interviews, and community pandemic committee meetings [36,38]. The qualitative data collected from the community engagement process guided the modifications to each community’s 1^st^ generation pandemic plan (Figure 1).

#### *Semi-directed interviews*

A round of interviews were conducted by the authors from February 9 to 23, 2010, with participants (n = 13) who were purposively chosen from each study community according to their government (i.e., local, provincial, or federal) role during their community’s response to pH1N1 (Table 1). The purpose of the interviews was to gain retrospective insight into the relationship between what was outlined in the community’s pandemic plan and what their pandemic response actually was [14]. In order to understand the barriers participants faced and suggested improvements for the pandemic response, the interview questions were based on the aspects of a health sector pandemic response outlined in academic literature [7,14]. Interviews were semi-directed in nature; thus, open-ended questions and frequent probes were used, allowing for participants to expand on points they perceived to be noteworthy in a confidential setting [39]. Each interview was conducted in English (as requested by the participants) at a time and place convenient for them. Additionally, upon consent, notes and audio recordings of the interviews were taken.

**Table 1:** Participants purposively selected for semi-directed interviews

	**Federal**	**Provincial**	**First Nations Band Council**
**Interviewed Participants**	- Health Director / Supervisor - Nurse-in-Charge - Community Health Nurse	- Director of Patient Care - Clinical Coordinator	- Chief - Deputy Chief

Interviews were manually transcribed verbatim and analyzed using a combination of deductive and inductive thematic coding by the author (NAC) and confirmed by the co-author (LJST) [40,41]. Following a template organizing approach, the seven sections of the First Nations and Inuit Health Branch – Ontario Region Pandemic Influenza Plan (FNIHB-OR PIP) were used as a coding template to deductively code data [42,43]. After reviewing the interview transcripts, segments of data relating to supplies and equipment (previously coded as ‘health services’) emerged as a new code (called ‘supplies’) via inductive coding [44].

The resulting eight codes formed the framework for each community’s 2^nd^ generation pandemic plan. Different colours were used as an organizational tool to highlight proposed modifications ascertained from the collected data suggested by the following: participants from their respective community; participants from the other two study communities; and relevant academic literature. Since all communities shared similar living conditions, other study communities’ suggestions were also presented as these may have been relevant to the other communities.

#### *Unstructured interviews*

Modified unstructured interviews were conducted by the author (NAC) from May 31 to June 10, 2010, with the health director/supervisor and/or nurse-in-charge (n = 4) of each study community’s health center, as he/she assumed a lead role during the public health emergency response and had the authority to comment on all aspects of the community’s pandemic response. During the interviews, each page of the 2^nd^ generation pandemic plan was reviewed, which provided the opportunity for participants to review and discuss the proposed colour-coded modifications. The interviews were conversational in nature, and employed follow-up and specifying questions [45]. For instance, questions were asked to obtain additional details about the community’s alternate care site (i.e., opened sites which provide supplementary primary health care and treatment) plan and morgue plan [7]. Desired changes were addressed, resulting in the 3^rd^ generation pandemic plan.

#### *Community pandemic committee meetings*

Each of the study communities had formed a community pandemic committee which comprised of community stakeholders involved in the community’s pandemic response. To engage participation from the community members, the 3^rd^ generation pandemic plan was subsequently presented to each study community’s pandemic committee (n = 27) for them to view during the period from July 27 to August 13, 2010. Each meeting was attended by eight to ten representatives from the following: health center, provincial hospital, nursing station, Band Council, education, clergy, Northern (a store), water treatment plant, and emergency medical services.

The meetings were facilitated by the author (NAC) to provide the forum for committee members to review, validate, and discuss the contents of each page of their community’s pandemic plan. In addition to each member receiving a personal copy of the pandemic plan during the meeting, a computer projector was used to display the plan and track-changes were used as an organizational tool to highlight the committee’s feedback. The resulting 4^th^ generation pandemic plan is the current version in place; therefore, multiple copies were distributed among committee members. Additionally, selected representatives (e.g., Chief, health director/supervisor) were given digital versatile discs (DVDs) of their respective community’s pandemic plan; thereby, allowing the dynamic plan to be accessed and changed accordingly to the community’s future needs.

## Results

The modifications to the study communities’ pandemic plans were made by the author (NAC) after each round of citizen input (i.e., semi-directed interviews, unstructured interviews, and community pandemic committee meetings) and the primary ones are highlighted below (Table 2).

**Table 2:** Summary of primary modifications made to community-level pandemic plans during the community engagement process

**Category**	**Modifications Made**
Surveillance	Added that health staff are responsible to monitor absenteeism in schools and workplaces.
Antivirals and Antibiotics	Added details of how antivirals are transported, received, stored, and who to contact when more medication is required.
Health Services	Added details about influenza-like illness (ILI) screening at healthcare facilities. Added that community health nurses are to provide basic personal protective equipment and self-care training. Added details about the home support program.
Supplies (added category)	Included information about ordering, maintaining, and providing influenza pandemic supplies.
Appendix (added section)	Added details outlined in Table 3.

### 1^st^ Generation pandemic plan

Each study community’s existing 1^st^ generation pandemic plan closely resembled the FNIHB-OR PIP, although some revisions were made prior to their response to pH1N1 [46]. These revisions were made after some representatives from the Band Council and health center attended pandemic meetings and tabletop exercises. For instance, in some cases, community-based people were assigned specific roles during the pandemic response and appropriate locations in the community were identified (e.g., where to establish an alternate care site, where to store vaccines, etc.). However, more information in the plans was required, as one participant mentioned:

"… there was a template … we work[ed] on that, it was done back in 2006, I believe at first, but it was never followed up on, it was not finished thoroughly … then we made … additional recommendations here and there, we added some things that need be … but it was also a learning process for us, because … there were some things that didn’t work or we would just kind of improvise … (Participant # 13)."

In general, the communities’ pandemic plans were divided into three phases (e.g., pre-pandemic, pandemic, and post-pandemic). Each phase was further divided into the seven categories of preparedness and response (e.g., surveillance, vaccine, antivirals and antibiotics, health services, emergency response, public health measures, and communications). Each category included details of what tasks the community was responsible for, who was responsible to complete the tasks, and when the tasks were to be completed by.

### 2^nd^ Generation pandemic plan

In general, the framework of the communities’ 2^nd^ generation pandemic plans remained similar; however, ‘supplies’ was added as a category in each phase, consistent with it being an emerging code from the data. The primary modifications which resulted in the 2^nd^ generation pandemic plans involved adding community-specific details. The ‘health services’ category included more details about influenza-like illness (ILI) screening at healthcare facilities, such as, identifying alternate entrances and waiting areas to isolate ILI cases from non-ILI cases, and guidelines to follow. Also, it was added that the community health nurses are responsible for providing basic personal protective equipment (PPE) training (e.g., how to wear masks, gowns, gloves, goggles, etc.) for staff at the health care facilities and community members when needed. Participants from the study communities reported that there was a lack of supplies for their pandemic response and ordered supplies did not arrive in a timely fashion. Thus, the new ‘supplies’ category included information about ordering, maintaining, and providing influenza pandemic supplies. Participants requested that additional information be included in some categories (e.g., vaccines, supplies, public health measures, and communications); therefore, it was later decided to include this information in an Appendix section in the 3^rd^ generation pandemic plan (Table 3).

**Table 3:** **List and description of appendices included in 3**^**rd**^**generation pandemic plans**

**Title of Appendix**	**Description**
World Health Organization Pandemic Influenza Phases	Describes the pandemic influenza phases (1–6), post-peak period, and post-pandemic period [52].
Canadian Activity Level	Describes Canadian Activity Levels 0, 1, and 2 [53].
Mass Immunization Clinic Protocol	Describes how to prepare and implement a community mass immunization clinic, including members involved and main responsibilities (e.g., vaccine safekeeping, staffing, orientation/training required, safety and security, communication, vaccinating community, and post-clinic issues) [54].
Public Health Agency of Canada: Mass Immunization Clinics in Remote & Isolated Communities	Provides the website link for more information about how to implement a mass immunization clinic specifically in remote and isolated communities [54].
Human Resource Contingency Plans	Includes human resource contingency plans for workplaces in the community.
List of Essential Services	Lists services in the community that are considered “essential” and must be maintained throughout a public health emergency.
Alternate Care Site Plan	Lists the organizers responsible for establishing an alternate care site, and possible locations in the community.
Outbreak Control Team and Clinical Pandemic Response Group/Command Center	Lists the members and responsibilities of the community outbreak control team and clinical pandemic response group/command center.
List of Pandemic Influenza Supplies	Lists general pandemic influenza supplies that are recommended to have available for the community.
First Nations and Inuit Health Branch Formulas for Pandemic Influenza Supplies	Provides formulas for calculating how many pandemic influenza supplies should be ordered for healthcare facilities and workplaces in the community.
Emergency Preparedness and Response Plan	Includes the community emergency preparedness and response plan.
Corpse Storage and Temporary Morgue Plan	Includes the community corpse storage and temporary morgue plan for summer and winter scenarios.
Community Infection Control Measures	Describes recommended community infection control measures to mitigate a pandemic (both mild and severe scenarios) [53]. Included components: public education, infection control measures, where to access health care, general comfort measures, isolation measures, quarantine measures, travel restrictions, screening measures, supplies, communication, closing schools/workplaces, and restricting public gatherings [53].
Templates for Community Notices	Includes templates for community notices (both mild and severe scenarios) that can be modified as needed.
Communication Plan	Outlines the community’s communication plan before, during, and after a pandemic. Describes various methods that can be used to communicate with community members, and the roles of the health center, community pandemic committee, and Band Council.
Helpful Resources	Provides website links (e.g., Health Canada, Public Health Agency of Canada, World Health Organization, etc.) for more information, which was also included in a DVD format for easier access to the information [2,7,43,53,54].

### 3^rd^ Generation pandemic plan

In general, after the unstructured interviews, more community-specific detail was incorporated into the communities’ pandemic plans. In the ‘surveillance’ category, it was added that the health staff are responsible to monitor absenteeism in schools and workplaces on a weekly basis during the regular influenza season and on a daily basis if ILI cases in the community increase by ten percent. Some participants reported that there was confusion about which health care facility was responsible for receiving and distributing antivirals. Thus, in the ‘antivirals and antibiotics’ category, specific detail of how antivirals are transported, received, stored, and who to contact when more medication is required was added. Furthermore, participants from all of the study communities stated that there was a general lack of community awareness during the pandemic response. As mentioned by one participant:

"…we didn’t really get all the education background … until the very last minute … they needed more awareness about it … especially in the school system, cause the kids don’t really understand … (Participant # 7)."

Thus, in the ‘health services’ category, it was recommended for the community health nurses to teach self-care training topics (e.g., general infection control measures and influenza education, etc.) at the school and workplaces and for all other community members. Also, details of the home support program, which would provide supplies and resources for ill families, were included. The main modification to highlight was the added Appendix section, which included sixteen appendices with detailed supplemental information to guide the community’s pandemic response (Table 3).

### 4^th^ Generation pandemic plan

After the community pandemic committee meetings, some noteworthy changes were made to the Appendix section, which were similar for all three communities. Information regarding the organizers, locations, and special considerations was added in the alternate care site plans. Furthermore, a participant had questioned:

"… the timing of this, the virus, if it happened like, in the spring, our springs are usually nice and warm, and where would we store the bodies? (Participant # 10)."

Thus, potential locations for a morgue in the community were chosen for summer and winter influenza pandemic scenarios and included in the corpse storage and temporary morgue plan. In Community C’s communication plan, it was added that, if necessary, practitioners of traditional First Nations medicine will provide health teachings.

## Discussion

### Community engagement process

Our research employed a community engagement process which involved (semi-directed and unstructured) interviews and collaborative debriefing and planning meetings. By comparing the communities’ 1^st^ and 4^th^ generation pandemic plans, our results suggest that there was a vast difference between what was initially outlined in the plans and what their response actually comprised of. Most of the resulting modifications incorporated into the study communities’ pandemic plans involved the addition of community-specific details and the clarification of the roles and responsibilities of involved organizations. Our results indicate that it was essential to the communities to add details of how, when, where, and who was responsible to implement recommendations outlined in the pandemic plans. Also, due to the geographical remoteness of the study communities, it was important to include details about supplies and resources in a newly created category. Interestingly, our results indicate that each of the communities added similar information (with slight variations) to their pandemic plans, which may be attributable to the fact that all are remote and isolated First Nations communities. This finding may reflect that while adding community-specific information to pandemic plans is important, it is also of great value to generally address the unique conditions of a region.

As a result of the community engagement process many modifications were made to the communities’ pandemic plans; however, some areas still require attention in the near future. For instance, as recommended by the participants, human resource contingency plans for each workplace in the community should be included. Also, the community infection control measures should be updated according to emerging research, especially regarding which pandemic mitigation measures are effective in remote and isolated communities. Since community-level pandemic plans are dynamic in nature, it is recommended that the plans are re-assessed and modified with community participation on an annual basis and after each public health emergency in order to meet the evolving needs of the community.

Our results indicate that the community engagement process had numerous benefits, in addition to eliciting valuable community-based input. For instance, the process employed provided the forum for the community pandemic committee to debrief about their pH1N1 response and to plan accordingly for the next public health emergency, especially with regards to discussing culturally sensitive issues, such as morgue locations. This group forum also provided the opportunity for committee members to clarify and be more cognizant of their respective roles and responsibilities. Furthermore, since the included participants represented various community stakeholders involved in the community’s pH1N1 response and multiple qualitative methods were used, the process gained insight into the diverse perspectives of community members living in remote and isolated communities [47].

Amongst the numerous benefits of the study’s employed community engagement process, some limitations were noted. First of all, we assumed that the included participants were reliable sources of information about the topic [22]. However, information from relevant academic literature was also included to supplement the communities’ opinions. Secondly, the process was labour intensive and expensive to conduct and thus, may not be realistically implemented on a large scale. Nevertheless, to elicit community participation, policy planners are encouraged to implement selected aspects of the presented process that they deem to be feasible. Thirdly, some of the modifications incorporated into the study communities’ pandemic plans may not be widely generalizable to the broader population due to the unique characteristics of the study communities [14].

### Recommendations

Our research has various implications for pandemic policy planners at all levels (i.e., community, regional, provincial, and federal). Although incorporating expert knowledge is imperative for pandemic planning at the national level, flexibility is required at the community-level to allow for plans to be adapted to address communities’ realities [17-20]. Research has displayed the importance of addressing local and culturally important beliefs and values in pandemic plans to ensure community acceptance, support, and compliance [2,21,22,47,48]. This is particularly important for First Nations living in remote and isolated communities due to their unique characteristics which may impact their ability to respond to a public health emergency [4-10]. The community engagement process that we employed demonstrates that effectively engaging the community in pandemic planning is possible in a relatively short time period [48]. Similar to the findings from other studies, our results suggest that community members possess a wealth of information from their personal experiences and can provide invaluable insight about local values and beliefs regarding public health emergencies [22,48].

Many methods exist to engage the public in pandemic planning (e.g., surveys, qualitative methods, deliberative forums, and social media); however, there is no consensus on how public engagement should be acquired as each method has its benefits and limitations [18,21,22,24,26,48-51]. For instance, surveys and qualitative methods (e.g., focus groups, interviews) have been shown to inform policy decision-making by providing public opinion about recommendations; however, a concern is that the public may not completely understand the complexity of the issue(s) [18,21,22]. Thus, to counteract the concern of the public having a limited understanding of the issue, deliberative forums can be used in conjunction as the participants are provided with detailed information prior to eliciting their opinion. Therefore, to offset the limitations and leverage the benefits of methods used, we recommend using multiple methods of public engagement in a complementary fashion to gain a broader understanding of a community’s views with regards to pandemic planning in order to produce the most effective plans [22].

## Conclusions

Overall, it is important that pandemic plans address local beliefs and values; therefore, it is imperative to engage citizens in pandemic planning as they can provide invaluable insight into local perspectives. Furthermore, it is especially important to engage and address concerns voiced by disadvantaged populations, such as First Nations living in remote and isolated communities, as they experience unique living conditions and are predicted to be disproportionately impacted by a public health emergency.

The employed community engagement process involved interviews and meetings, and resulted in eliciting and incorporating community-based input into the pandemic plans of three remote and isolated First Nations communities of sub-arctic Ontario, Canada. Our results indicate that the community engagement process successfully resulted in up-to-date, community-informed, and culturally-appropriate community-level pandemic plans. We recommend that this process be used alongside other methods of public engagement, in addition to expert consultation, to create the most effective plans. Since pandemic plans are dynamic in nature, the plans must be continually modified and updated to meet the evolving needs of each community. It is hoped that these modified pandemic plans will lay the foundation for an improved pandemic response in these remote and isolated First Nations communities. Furthermore, the suggested modifications may help inform updates to the community-level pandemic plans of other similar communities.

## Competing interests

The authors declare that they have no competing interests.

## Authors’ contributions

NAC contributed to the original concept and design of the study, literature review, data collection and analyses, and drafted the manuscript. LJST contributed to the original concept and design of the study, data collection and analyses, and manuscript drafts. All authors read and approved the final manuscript.

## Pre-publication history

The pre-publication history for this paper can be accessed here:

http://www.biomedcentral.com/1471-2458/12/268/prepub
